# Supplementary material for: Assessing Reproductive Performance to Establish Benchmarks for Small-Holder Beef Cattle Herds in South Africa
Source: Animals (Basel). 2022 Nov 1;12(21):3003. doi: 10.3390/ani12213003 (PMC9657001; doi:10.3390/ani12213003)
Supplement: Supplementary file 1 [file animals-12-03003-s001.zip › animals-1931623-supplementary.pdf]

Supplementary material

Table S1. Summary of the likelihood and variation of reproductive performance in smallholder beef cattle herds between provinces (Eastern Cape, Free State, Limpopo, Mpumalanga and North West).

Indicator	Estimate	Standard error	95% CI of OR Lower	Upper	P value	PP	Variation
**PD**					**0.0717**	**0.48**	**0.03**
Mpumalanga	0.5066	0.1538	0.2050	0.8081			
Eastern Cape	-0.03525	0.1550	-0.3391	0.2686			
Limpopo	-0.3704	0.1621	-0.6883	-0.05251			
North West	0.07386	0.1646	-0.2489	0.3966			
Free State	-0.1724	0.2045	-0.5734	0.2286			
**FL**					**0.0921**	**0.13**	**0.13**
Mpumalanga	0.2252	0.3608	-0.4829	0.9334			
Eastern Cape	-0.9643	0.3964	-1.7423	-0.1862			
Limpopo	0.9146	0.4028	0.1240	1.7052			
North West	0.3184	0.3960	-0.4589	1.0956			
Free State	-0.3833	0.4888	-1.3425	0.5760			
**DO**							
Mpumalanga	0.3936	0.2271	-0.05201	0.8392	**0.0926**	**0.83**	**0.27**
Eastern Cape	0.07890	0.2266	-0.3657	0.5235			
Limpopo	-0.07653	0.2412	-0.5497	0.3966			
North West	0.4069	0.2522	-0.08782	0.9017			
Free State	-0.8030	0.2893	-1.3705	-0.2354			
**CI**					**0.0923**	**0.92**	**0.06**
Mpumalanga	-0.1497	0.2271	-0.5952	0.2958			
Eastern Cape	-0.2402	0.2285	-0.6885	0.2082			
Limpopo	-0.4831	0.2539	-0.9811	0.01495			
North West	0.09089	0.2462	-0.3921	0.5738			
Free State	0.7949	0.2830	0.2398	1.3499			

Table S2. Cow characteristics

Characteristics	Frequency %
**Breed type**	
Nguni type	9.37
Afrikaner type	5.32
Angus type	3.62
Beef master type	15.28
Bonsmara type	39.85
Boran type	1.95
Brahman type	3.79

Drakensberger type	4.74
Hereford type	4.74
Hugenoot type	1.28
Simbrah type	3.65
Simmental type	6.41
**Age**	
**3**	3.18
**4**	12.85
**5**	27.43
**6**	25.31
**7**	19.85
**8+**	11.38
**Parity**	
1	31.64
2	31.93
3	21.55
4	12.48
5+	2.40
